# Appropriate Fe (II) Addition Significantly Enhances Anaerobic Ammonium Oxidation (Anammox) Activity through Improving the Bacterial Growth Rate

**DOI:** 10.1038/srep08204

**Published:** 2015-02-03

**Authors:** Yiwen Liu, Bing-Jie Ni

**Affiliations:** 1 Advanced Water Management Centre, The University of Queensland, St Lucia, Brisbane, Queensland 4072, Australia

## Abstract

The application of anaerobic ammonium oxidation (Anammox) process is often limited by the slow growth rate of Anammox bacteria. As the essential substrate element that required for culturing Anammox sludge, Fe (II) is expected to affect Anammox bacterial growth. This work systematically studied the effects of Fe (II) addition on Anammox activity based on the kinetic analysis of specific growth rate using data from batch tests with an enriched Anammox sludge at different dosing levels. Results clearly demonstrated that appropriate Fe (II) dosing (i.e., 0.09 mM) significantly enhanced the specific Anammox growth rate up to 0.172 d^−1^ compared to 0.118 d^−1^ at regular Fe (II) level (0.03 mM). The relationship between Fe (II) concentration and specific Anammox growth rate was found to be well described by typical substrate inhibition kinetics, which was integrated into currently well-established Anammox model to describe the enhanced Anammox growth with Fe (II) addition. The validity of the integrated Anammox model was verified using long-term experimental data from three independent Anammox reactors with different Fe (II) dosing levels. This Fe (II)-based approach could be potentially implemented to enhance the process rate for possible mainstream application of Anammox technology, in order for an energy autarchic wastewater treatment.

Anaerobic ammonium oxidation (Anammox) process is currently considered as one of the most sustainable alternatives to the conventional costly nitrification-denitrification biological nitrogen removal process^1^, particularly when the wastewater contains high nitrogen but only small amounts of biologically degradable carbon sources^2^. In autotrophic Anammox process, ammonium is directly oxidized to nitrogen gas utilizing nitrite as the electron acceptor^3^,^4^, without the need for organic carbon source as compared to heterotrophic denitrification^5^,^6^. Also, Anammox process largely reduces oxygen demand in the nitrification as the ammonium is only required to be nitrified to nitrite instead of nitrate^7^. Furthermore, the biomass yield of Anammox bacteria is very low, resulting in a small amount of excess sludge production and thus lower operational costs^8^,^9^. Overall, Anammox process can reduce oxygen demand by 64%, exogenous carbon source by 100% and sludge production by 80–90%^10^ as compared to conventional nitrogen removal process^11^.

However, the application of Anammox process is often limited by the slow growth rate of Anammox bacteria with a relative long doubling time of 7–20 d^12^,^13^. In addition, Anammox bacteria are very sensitive to operational conditions such as dissolved oxygen, temperature, pH and organic matters^14^,^15^. Unfavorable conditions would largely reduce Anammox activity, leading to process failure during application. Therefore, effectively enhancing Anammox activity to achieve a high rate process is a subject of great interest, especially for the application of Anammox process under mainstream conditions that might lead to large decrease in specific anammox activity^16^,^17^,^18^. A number of strategies have been developed, such as application of magnetic field, electric field and ultrasound^19^,^20^,^21^,^22^,^23^,^24^. However, most of these methods are cost intensive owing to high energy input with low feasibility in terms of large-scale application. Thus, alternative cost-effective approach to enhance Anammox activity is highly desired.

Ferrous iron (Fe (II)) is an important and essential substrate element for the growth of Anammox bacteria, which has been widely used for culturing Anammox sludge^5^. Several studies have demonstrated that iron could affect the metabolism of Anammox bacteria^19^,^25^. van Niftrik et al.^26^ found that Anammox bacteria would store iron inside Anammoxosome compartment in order to have an excess supply of iron for further Heme c synthesis. Thus, it could be expected that Fe (II) could have an impact on Anammox bacterial growth. However, since Van de Graff et al.^5^ firstly reported the discovery of Anammox process, Fe (II) concentration in the feeding solution of almost all the enriched Anammox sludge system was constantly set as 0.03 mM. Little efforts has been dedicated to understanding the effects and roles of different amounts of Fe (II) addition on Anammox activity^27^. In addition, currently well-established Anammox models do not consider the possible effects of Fe (II) on Anammox bacteria growth^28^,^29^,^30^. As such, the Anammox model is not able to predict Anammox dynamics caused by Fe (II) variations in real applications.

Therefore, this work aims to systematically investigate the effects of Fe (II) addition on Anammox activity based on the kinetic analysis of specific growth rate using previously reported data from extensive batch tests with an enriched Anammox sludge at different Fe (II) dosing levels. The relationship between Fe (II) concentration and specific Anammox growth rate was analysed and integrated into currently well-established Anammox model to describe the enhanced Anammox growth with Fe (II) addition. The validity of the integrated Anammox model was verified using long-term experimental data from three independent Anammox reactors with different Fe (II) dosing levels.

## Results

### The Effect of Fe (II) Addition on Anammox Activity

**Figure 1:**
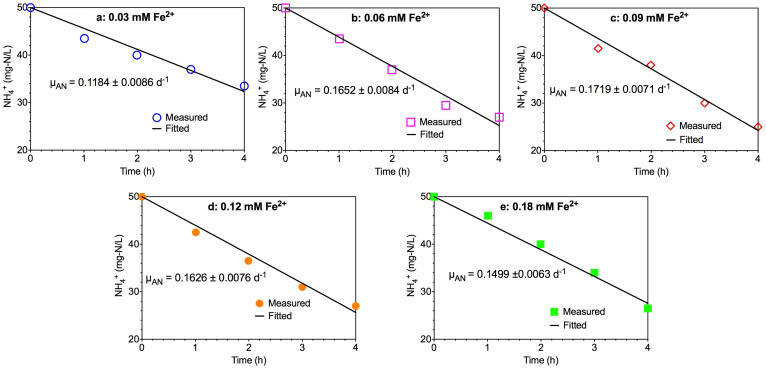
The experimental measured and kinetic fitted ammonium consumption profiles in five 4-h batch tests under different Fe (II) concentrations: (a) 0.03 mM; (b) 0.06 mM; (c) 0.09 mM; (d) 0.12 mM; and (e) 0.18 mM (symbols represent experimental measurements and lines represent kinetic fittings).

Figure 1 presents the measured and kinetic fitted ammonium consumption profiles from the five batch tests at different Fe (II) addition levels. The ammonium concentration decreased linearly in all the five batch experiments with different reduction rates. The kinetic fittings matched well with all the experimental measurements. In general, Fe (II) addition with a concentration higher than the normal level (0.03 mM) enhanced the Anammox activity in terms of ammonium consumption rate. The estimated specific Anammox growth rates (*μ_AN_*) in the five batch tests with standard errors are also shown in Figure 1. With the increase of Fe (II) concentrations from 0.03 mM (control experiment, Figure 1a) to 0.09 mM (Figure 1c), the estimated specific growth rates of Anammox microorganisms increased from 0.1184 d^−1^ to 0.1719 d^−1^. The obtained kinetic value of 0.1184 d^−1^ under normal Fe (II) condition (0.03 mM) was also comparable with literature reported values^28^. Further increasing Fe (II) concentration to 0.18 mM slightly decreased the specific Anammox growth rate to 0.1499 d^−1^ (Figure 1e). The results indicated that the highest specific Anammox growth rate was achieved at a Fe (II) concentration of 0.09 mM under the studied conditions, which was 45% higher than that in control test (Figure 1a). These results demonstrated that appropriate Fe (II) addition could significantly enhance the Anammox activity.

### Relationship between Fe (II) and Anammox Growth Rate

**Figure 2:**
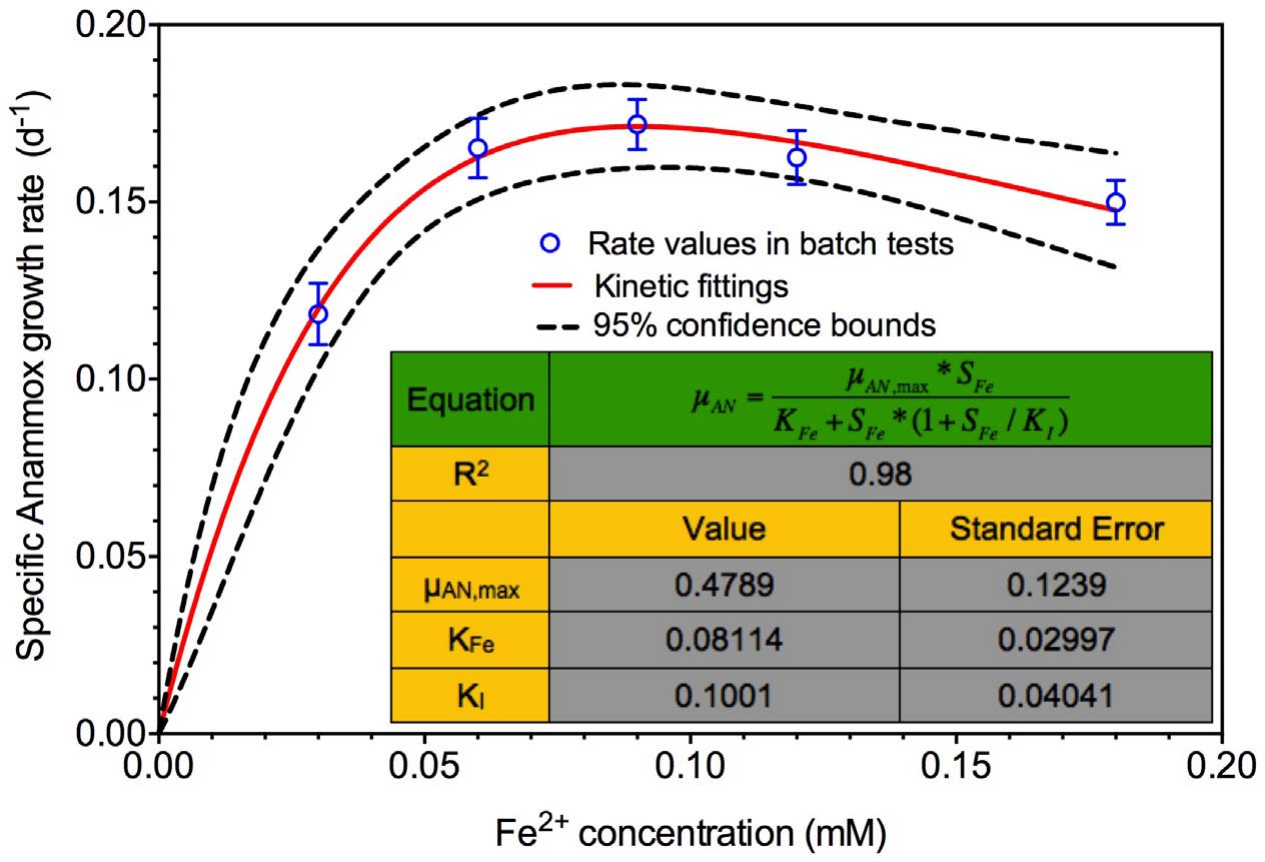
The experimentally observed and model-fitted relationship between Fe (II) concentrations and the specific Anammox growth rates using the substrate inhibition kinetics (symbols represent the rate values in batch tests, red line represents the kinetic fitting profile, and black dash represents the predicted 95% confidence bounds during the fitting).

The batch test results clearly showed that appropriate Fe (II) addition increased the specific Anammox growth rate but over-dosing Fe (II) slightly decreased the rate (Figure 1). Although Fe (II) is the essential substrate element that required for culturing Anammox sludge, it has been also reported that Fe (II) at a high concentration could likely induce biomass destruction^31^,^32^. Therefore, high concentrations of Fe (II) might lead to a slight inhibition on Anammox activity, as observed in this study. Figure 2 summarizes the estimated specific Anammox growth rates (*μ_AN_*) in all of the batch tests at each of the corresponding Fe (II) concentrations. The dependence of *μ_AN_* on the Fe (II) concentration could be well described using the typical substrate inhibition kinetics^33^ with R^2^ value of 0.98 as shown in Figure 2 and following equation.
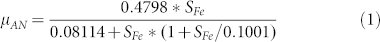
where 0.4798 d^−1^ is the maximum growth rate of Anammox bacteria under the studied conditions, 0.08114 mM is half-saturation concentration constant and 0.1001 mM is inhibition constant. The predicted 95% confidence bounds included all the data points, further indicating that the effect of Fe (II) on Anammox growth rates could be well described by substrate inhibition kinetics (red line, Figure 2), with the best Fe (II) dosing concentration at 0.09 mM under the studied conditions.

### Evaluating the Integrated Anammox Model with Fe (II) Effects

The evaluation on the proposed integrated Anammox model with Fe (II) effects (Equations 4–6, Method Section) and the obtained parameters regarding Fe (II) addition were based on the comparison between the model predictions using the parameter values in Equation 1 and independent experimental data collected from the 120-day operation of the three Anammox reactors under different Fe (II) dosing conditions, namely R1 (control, 0.03 mM Fe (II), 0–120 d), R2 (0.06 mM Fe (II) during 0–70 d and 0.12 mM Fe (II) during 71–120 d) and R3 (0.09 mM Fe (II) during 0–70 d and 0.18 mM Fe (II) during 71–120 d).

**Figure 3:**
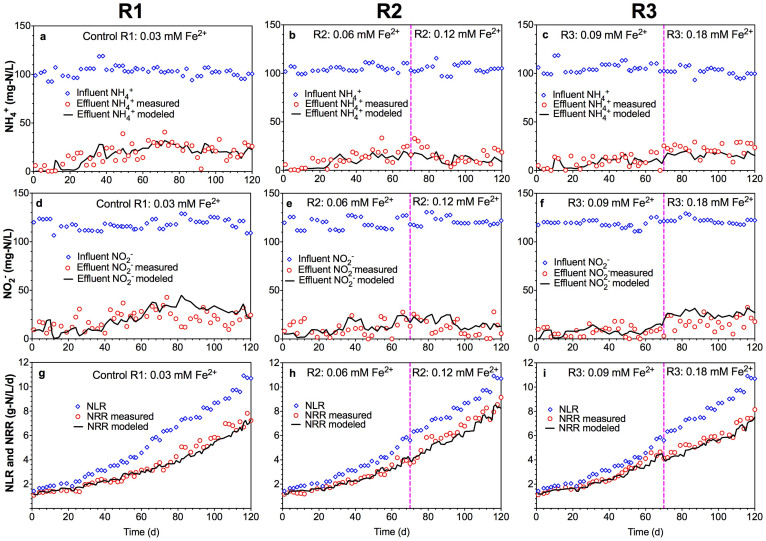
The measured and simulated ammonium (a–c), nitrite (d–f) and NRR (g–i) during long-term (120 days) continuous experiments in R1, R2 and R3, respectively (symbols represent experimental measurements and black lines represent model predictions using the integrated Anammox model of this work). The influent Fe (II) concentrations was 0.03 mM constantly in R1 (a, d and g), while it was 0.06 mM during 0–70 d and then increased to 0.12 mM between 71–120 d in R2 (b, e and h), and 0.09 mM during 0–70 d and then increased to 0.18 mM between 71–120 d in R3 (c, f and i), respectively.

The model predictions and the experimental results in terms of effluent ammonium, nitrite and nitrogen removal rate (NRR) are compared in Figure 3. The evaluation results showed that the model predictions matched all the measured data in the three independent Anammox reactors, which supported the validity of the integrated model and the parameters for describing enhanced Anammox activity by Fe (II) addition. In comparison, the model prediction without considering Fe (II) effect poorly fitted the experimental data (Figure S1). During the initial 70 days, R3 with a Fe (II) concentration of 0.09 mM exhibited lower ammonium and nitrite concentrations in the effluent than those in R2 with 0.06 mM Fe (II) and R1 with 0.03 mM Fe (II), resulting in a higher NRR (Figure 3). In contrast, during 70–120 days, R2 with a Fe (II) concentration of 0.12 mM showed better ammonium and nitrite removal performance than R3 with 0.18 mM Fe (II) and R1 with 0.03 mM Fe (II), suggesting the slightly decrease of Anammox activity under high Fe (II) concentration conditions.

**Figure 4:**
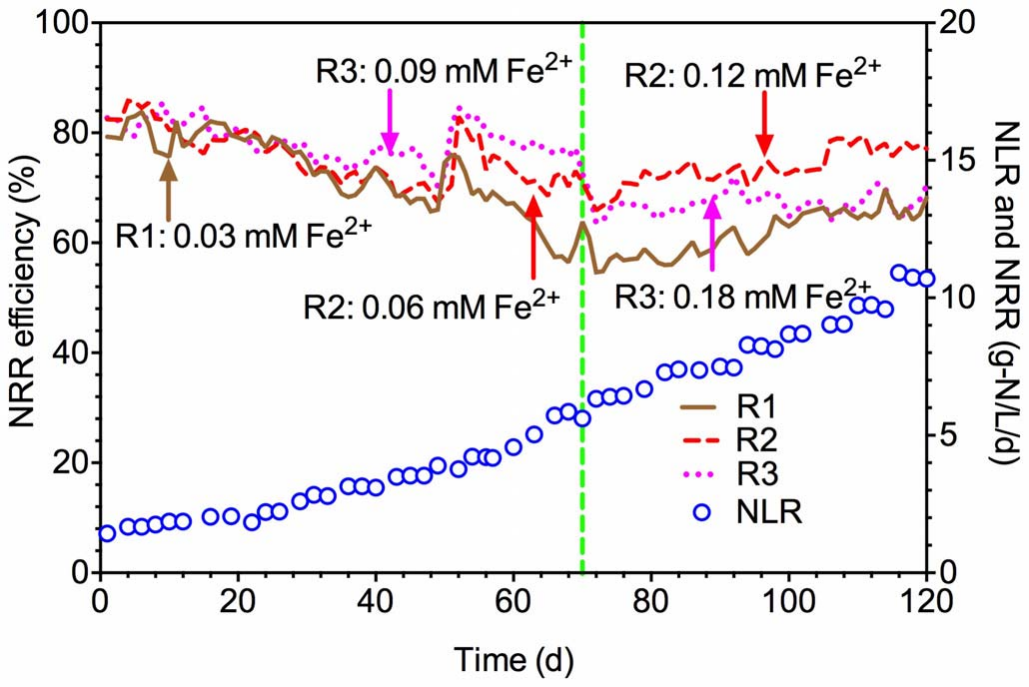
The model simulated NRR efficiency during long-term (120 days) continuous experiments in R1, R2 and R3, respectively. The influent Fe (II) concentrations was 0.03 mM constantly in R1, while it was 0.06 mM during 0–70 d and then increased to 0.12 mM between 71–120 d in R2, and 0.09 mM during 0–70 d and then increased to 0.18 mM between 71–120 d in R3, respectively.

To better compare the nitrogen removal performance in three different Anammox reactors, model predicted NRR efficiency was shown in Figure 4. At the low nitrogen loading rate (NLR) between 1.5 and 3 g-N/L/d, all three reactors showed similar performance. During 30–70 days with the NLR of 3–6 g-N/L/d, R3 exhibited higher NRR efficiency than those of R1 and R2. During 71–120 d, the NRR efficiency of R3 started to drop from ca. 70% to 65% due to the overdose of Fe (II). In contrast, R2 with a Fe (II) dose of 0.12 mM at this stage showed the highest NRR efficiency among all three reactors. These results further confirmed that appropriate Fe (II) addition enhanced Anammox activity.

## Discussion

Recently, strategies to enhance Anammox activity for its full application in biological nitrogen removal have attracted more attentions due to the low growth rate of Anammox bacteria^19^,^20^,^22^. Among them, Fe (II) addition would be a novel and promising strategy for cost-effective enhancement of Anammox activity since Fe (II) is the essential substrate element that required for culturing Anammox sludge^27^. However, the effect of Fe (II) on Anammox activity has not been fully elucidated before. In this study, a kinetic analysis of the specific Anammox growth rate of an enriched Anammox sludge was performed using experimental data from extensive batch tests at different Fe (II) dosing levels. The operational conditions (e.g. NH_4_^+^, NO_2_^−^ and Anammox biomass) that would affect specific growth rate were controlled at same levels in each batch test. As a result, the effect of Fe (II) on Anammox activity was studied with minimum interference from other affecting factors, which revealed, for the first time, a substrate inhibition relationship between the specific Anammox growth rate and Fe (II) concentration (R^2^ = 0.98).

This direct dependency of specific Anammox growth rate on Fe (II) might be due to the important role of Fe (II) in linking energy generating process and cell growing process in Anammox metabolism^34^,^35^,^36^, although the fundamental mechanism is unclear at present. In Aanammox, hydrazine is an energy-rich compound, which can donate its electrons to produce reduced ferredoxin^37^. The four high-energy electrons from ferredoxin can then be used for energy conversion and generation of a proton motive force (PMF)^37^. The PMF would be used to energize an ATPase that is localized in the anammoxosomal membrane to produce ATP in the riboplasm^37^. The reduced ferredoxin can also act as an electron donor for carbon dioxide fixation in the acetyl-CoA pathway for Anammox growth^37^. In addition, evidence also showed that Fe (II) is an important element in the electron transport chains for the generation of Heme c, which is considered as an indispensable component of some key functional enzymes for the growth of Anammox bacteria^27^,^38^. Therefore, Fe (II) addition could be a potential approach to enhance the growth of Anammox bacteria, as demonstrated in this work (Figure 2). It is likely that the variations of Fe (II) concentrations could change the overall carbon assimilation rate and in turn affect ATP production/consumption that is coupled to the energy generating process of Anammox. This hypothesis remains to be verified.

Previous studies on Anammox activity mainly focused on its correlation to operational conditions including temperature, NO_2_^−^ accumulation, pH, and DO concentrations^14^,^15^. However, the effect of Fe (II) concentrations in real wastewater was neglected when applying Anammox process for full-scale wastewater treatment. The Fe (II) concentrations might vary largely among different municipal or industrial wastewater. The substrate inhibition kinetic relationship between Anammox growth rate and Fe (II) concentration revealed in this study (Figure 2) indicated that Fe (II) could be another important affecting factor accounting for the variability of Anammox activity in previous full-scale Anammox plants^39^.

Mathematical modeling has been widely applied to predict biological nitrogen removal processes including Anammox process^28^,^29^,^30^. However, currently well-established Anammox models do not consider the possible effects of Fe (II) on Anammox bacteria growth^28^,^29^,^30^. As such, these previous Anammox models are not be able to predict Anammox dynamics caused by Fe (II) variations. In this work, the substrate inhibition kinetic relationship between Fe (II) concentration and specific Anammox growth rate was integrated into currently well-established Anammox model to describe the enhanced Anammox growth with Fe (II) addition, which would potentially contribute to a more powerful mathematic tool that predicts the dynamics of Anammox activity more thoroughly. The validity of the new integrated Anammox model was successfully verified using long-term experimental data from three independent Anammox reactors with different Fe (II) dosing levels, supporting its applicability in such Anammox systems. It should be noted that the potential existence of heterotrophic bacteria was not considered in the current model. This is acceptable due to the fact that all the experiments were conducted under the condition without external organic carbon supply, which largely limited the growth of heterotrophs. Although biomass decay products or soluble microbial products may be used as organic carbon, heterotrophic growth on these sources was very small compared to the dominating Anammox microorganisms in the enriched Anammox sludge. However, the heterotrophic processes could be easily incorporated into the model, if heterotrophic activity would be included in the system. In addition, the successful application of the integrated model in this work confirmed the optimal Fe (II) dosing levels for Anammox growth could range between 0.06 and 0.12 mM.

Anammox process has been successfully implemented in sidestream treatment at wastewater treatment plant (WWTP) to reduce energy and carbon input for high-strength nitrogen removal^40^,^41^. Research focus has now moved to possible Anammox application into mainstream treatment in order to maximize energy and material savings^16^,^17^. Compared to sidestream wastewater treatment usually operated at temperatures exceeding 25°C, mainstream wastewater has a lower temperature range (10–25°C) as well as much lower nitrogen concentration. These conditions would largely reduce Anammox activity and thus decrease the process performance^42^. Based on the findings of this work that appropriate Fe (II) addition could effectively enhance Anammox activity, a new Fe (II)-based approach could be potentially implemented to enhance the process rate for possible mainstream application of Anammox technology. This strategy would be likely to counteract the decrease of Anammox activity resulting from low temperature, although this still warrant further experimental verification.

In summary, the effects of Fe (II) addition on Anammox activity was investigated based on the kinetic analysis of specific growth rates in extensive batch tests with an enriched Anammox sludge at different Fe (II) dosing levels. The results demonstrated that appropriate Fe (II) addition could significantly enhance Anammox activity through improving the bacterial growth rate. The relationship between Fe (II) concentration and specific Anammox growth rate was found to be well described by typical substrate inhibition kinetics, which was integrated into currently well-established Anammox model to describe the enhanced Anammox growth with Fe (II) addition. The validity of the integrated Anammox model was verified using long-term experimental data from three independent Anammox reactors with different Fe (II) dosing levels. This Fe (II)-based approach could be potentially implemented to enhance the process rate for possible mainstream application of Anammox technology, in order for an energy autarchic wastewater treatment.

## Methods

### Batch Experimental Data for Kinetic Analysis

Five sets of batch experimental data with different amounts of Fe (II) addition using an enriched Anammox sludge previously reported in Qiao et al. 27 were used for the kinetic analysis of Anammox process. The enriched Anammox sludge was acquired from a laboratory-scale Anammox reactor. Anammox bacteria of KSU-1 strain accounted for the majority of the total active biomass in this enriched Anammox sludge. Batch experiments were carried out in five 120 mL serum bottles containing same amount of substrate (ammonium and nitrite) and Anammox biomass but with different Fe (II) concentrations (details in Table 1). The media solution used in the batch experiments mainly consisted of ammonium and nitrite in the form of (NH_4_)_2_SO_4_ and NaNO_2_, respectively. The detail composition of the trace mineral medium was as described by Van de Graff et al. 5 except Fe (II) concentrations. The mixed liquor volatile suspended solids (MLVSS) concentration was about 1850 mg/L in each bottle. The pH was adjusted to 7.5 and the temperature was maintained at 35 ± 1°C in all batch experiments. The serum bottle contents were purged with nitrogen gas to remove dissolved oxygen. The initial NH_4_^+^-N and NO_2_^−^-N concentrations were both set at 50 mg-N/L at all five batch tests. Samples were taken from the serum bottles at 0, 1, 2, 3 and 4 h to analyze the ammonium consumption profiles.

### Kinetic Analysis of Specific Anammox Growth Rates

The typical kinetic model describing the anaerobic consumption rate of ammonium (*r_NH4_*) by Anammox bacteria (*X_AN_*) was shown in following equation with a Monod-based kinetics for Anammox process being used in this work.
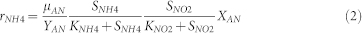
where *μ_AN_*is the specific growth rate (d^−1^) of Anammox bacteria that might vary with Fe (II) addition; *K_NH4_* and *K_NO2_* are ammonium and nitrite affinity constants for Anammox bacteria, with reported values being 0.73 and 0.55 mg-N/L^30^, respectively; *S_NH4_* and *S_NO2_* represent for ammonium and nitrite concentrations (mg-N/L), respectively; and Y_AN_ is the biomass yield of Anammox bacteria, with typical value being 0.159 mg-COD/mg-N^30^.

To investigate the effect of Fe (II) on Anammox activity, the five batch test results were used to estimate the specific Anammox growth rates (*μ_AN_*) at the five different Fe (II) concentration levels. The *μ_AN_* values were estimated by minimizing the sum of squares of the deviations between the measured ammonium concentrations and the model predictions using the secant method embedded in AQUASIM 2.1d^43^. The cell loss attributed to the endogenous decay over the 4-h batch experimental course was extremely small. Therefore, the biomass lose in the decay was neglected during the parameter estimations.

### Integrated Anammox Model Considering Fe (II) Effects

Based on the kinetic analysis results of the specific Anammox growth rate variations at different Fe (II) levels, a substrate inhibition kinetics was proposed and tested to describe the relationship between Fe (II) concentrations and specific Anammox growth rates as shown in following equation^33^.
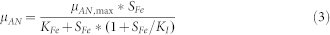
where *μ_AN_* and *μ_AN,max_* are the specific and maximum growth rate of Anammox bacteria, respectively; *S_Fe_* is Fe (II) concentration (mM); and *K_Fe_* and *K_I_* are half-saturation concentration and inhibition constant for Fe (II) (mM), respectively.

The obtained substrate inhibition kinetics describing the effects of Fe (II) was then incorporated into currently well-established Anammox model^28^,^30^ to develop an integrated Anammox model being able to describe the enhanced Anammox growth with Fe (II) addition. The kinetic equations of Anammox growth, ammonium consumption and nitrite utilization processes in the integrated model could be expressed as follows: 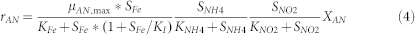

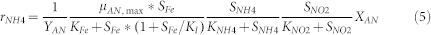

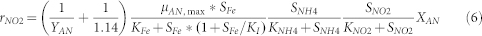
where *r_AN_*, *r_NH4_* and *r_NO2_* are the growth rate (mg-COD/L/d), ammonium consumption rate (mg-N/L/d) and nitrite utilization rate (mg-N/L/d) of Anammox bacteria that would vary at different Fe (II) addition levels. The endogenous decay process of Anammox bacteria is the same with previous Anammox models^30^.

### Long-Term Experimental Data to Verify the Integrated Model

In order to verify the validity of the integrated Anammox model describing the effects of Fe (II) dosing on Anammox activity, long-term continuous experimental data from three independent Anammox reactors with different Fe (II) dosing levels reported in Qiao et al.^27^ were used to evaluate the integrated model. Three identical upflow fixed-bed column reactors R1 (control), R2 and R3 were operated for 120 days continuously. The working volume of each reactor was about 0.3 L. Each reactor was inoculated with 30 g (wet weight) enriched Anammox sludge (same sludge to the batch experiments), resulting in an initial MLVSS of 3683 mg/L. All three reactors were continuously fed with the same media containing about 100 mg-N/L ammonium and 130 mg-N/L nitrite except for the Fe (II) concentrations (details in Table 1). The influent was purged with 99.5% N_2_ to prevent Fe (II) from being oxidized. R1 was continuously fed with 0.03 mM Fe (II) for 120 days as control, while R2 was fed with 0.06 mM Fe (II) during 0–70 d and then 0.12 mM Fe (II) between 71–120 d, and R3 was fed with 0.09 mM Fe (II) during 0–70 d and then 0.18 mM Fe (II) between 71–120 d, respectively.

The influent NLR of each reactor was gradually increased from 1.5 to 11 g-N/L/d by increasing the influent flow rate during the 120-day operation. The pH of the influent was adjusted to 7.0 and the temperature was maintained at 35 ± 1°C for all the three Anammox reactors. Effluent samples were taken from each Anammox reactor to analyze the ammonium and nitrite concentrations as well as to calculate the total nitrogen removal rate (NRR) during the 120-day operation. The experimental ammonium, nitrite and NRR data from the three Anammox reactors were compared with the model predictions using the parameter values obtained from batch tests (without further calibration) to verify the applicability of the integrated model describing the effects of Fe (II) on Anammox activity.

## Author Contributions

Y.L. and B.-J. N. wrote the manuscript; Y.L. and B.-J. N. developed the methodology; Y.L. performed data analysis and prepared all figures; Both authors reviewed the manuscript.

## Supplementary Material

Supplementary Information Supplementary material for Appropriate Fe (II) Addition Significantly Enhances Anaerobic Ammonium Oxidation (Anammox) Activity through Improving the Bacterial Growth Rate

## Figures and Tables

**Table 1:** Fe (II) Concentrations Used in Batch and Continuous Experiments

Experimental Design	Fe (II) concentrations (mM)	
**Batch Experiments**		
Batch test 1	0.03	
Batch test 2	0.06	
Batch test 3	0.09	
Batch test 4	0.12	
Batch test 5	0.18	
**Continuous Experiments**	Phase I (0–70 days)	Phase II (71–120 days)
Anammox reactor R1	0.03	0.03
Anammox reactor R2	0.06	0.12
Anammox reactor R3	0.09	0.18
