# Supplementary material for: Appropriate Fe (II) Addition Significantly Enhances Anaerobic Ammonium Oxidation (Anammox) Activity through Improving the Bacterial Growth Rate
Source: Sci Rep. 2015 Feb 3;5:8204. doi: 10.1038/srep08204 (PMC4316192; doi:10.1038/srep08204)
Supplement: Supplementary Information — Supplementary material for Appropriate Fe (II) Addition Significantly Enhances Anaerobic Ammonium Oxidation (Anammox) Activity through Improving the Bacterial Growth Rate [file srep08204-s1.pdf]

Supplementary material for

Appropriate Fe (II) Addition Significantly Enhances Anaerobic Ammonium Oxidation (Anammox) Activity through Improving the Bacterial Growth Rate

Yiwen Liu, Bing-Jie Ni*

Advanced Water Management Centre, The University of Queensland, St Lucia, Brisbane, Queensland 4072, Australia

***Corresponding author:**

Bing-Jie Ni, P +61 7 33463230; F +61 7 33654726; E-mail: b.ni@uq.edu.au

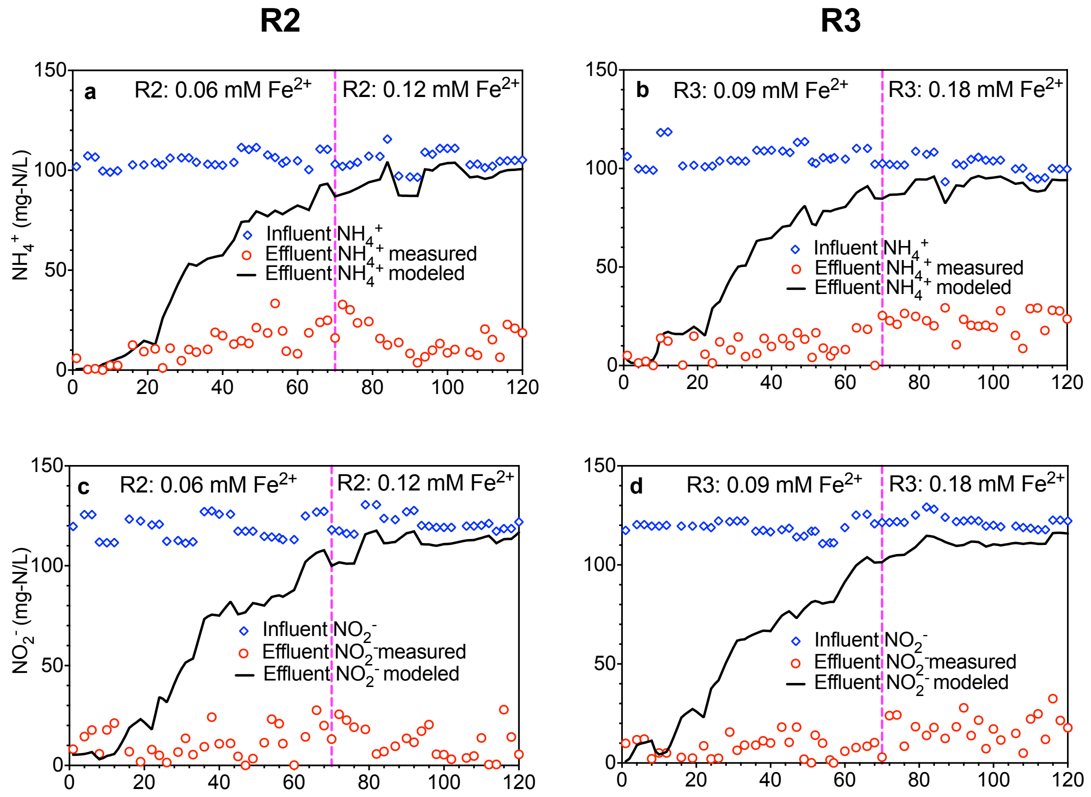

Figure S1 The measured and simulated ammonium (a, b) and nitrite (c, d) during long-term (120 days) continuous experiments in R2 and R3, respectively (symbols represent experimental measurements and black lines represent model predictions using the Anammox model without considering Fe (II) effect).
